# Horizontal Gene Transfer Regulation in Bacteria as a “Spandrel” of DNA Repair Mechanisms

**DOI:** 10.1371/journal.pone.0001055

**Published:** 2007-10-24

**Authors:** Saliou Fall, Anne Mercier, Franck Bertolla, Alexandra Calteau, Laurent Gueguen, Guy Perrière, Timothy M. Vogel, Pascal Simonet

**Affiliations:** 1 Environmental Microbial Genomics Group, Laboratoire AMPERE UMR CNRS 5005, Ecole Centrale de Lyon et Université de Lyon, Ecully, France; 2 Ecologie Microbienne, UMR CNRS 5557, Université Claude Bernard–Lyon 1, Villeurbanne, France; 3 Laboratoire de Biométrie et Biologie Évolutive, UMR CNRS 5558, Université Claude Bernard–Lyon 1, Villeurbanne, France; Oxford University, United Kingdom

## Abstract

Horizontal gene transfer (HGT) is recognized as the major force for bacterial genome evolution. Yet, numerous questions remain about the transferred genes, their function, quantity and frequency. The extent to which genetic transformation by exogenous DNA has occurred over evolutionary time was initially addressed by an *in silico* approach using the complete genome sequence of the *Ralstonia solanacearum* GMI1000 strain. Methods based on phylogenetic reconstruction of prokaryote homologous genes families detected 151 genes (13.3%) of foreign origin in the *R. solanacearum* genome and tentatively identified their bacterial origin. These putative transfers were analyzed in comparison to experimental transformation tests involving 18 different genomic DNA positions in the genome as sites for homologous or homeologous recombination. Significant transformation frequency differences were observed among these positions tested regardless of the overall genomic divergence of the *R. solanacearum* strains tested as recipients. The genomic positions containing the putative exogenous DNA were not systematically transformed at the highest frequencies. The two genomic “hot spots”, which contain *recA* and *mutS* genes, exhibited transformation frequencies from 2 to more than 4 orders of magnitude higher than positions associated with other genes depending on the recipient strain. These results support the notion that the bacterial cell is equipped with active mechanisms to modulate acquisition of new DNA in different genomic positions. Bio-informatics study correlated recombination “hot-spots” to the presence of Chi-like signature sequences with which recombination might be preferentially initiated. The fundamental role of HGT is certainly not limited to the critical impact that the very rare foreign genes acquired mainly by chance can have on the bacterial adaptation potential. The frequency to which HGT with homologous and homeologous DNA happens in the environment might have led the bacteria to hijack DNA repair mechanisms in order to generate genetic diversity without losing too much genomic stability.

## Introduction

The fundamental impact of horizontal gene transfer (HGT) in shaping the structure of bacterial genomes was only recently demonstrated by the analysis of numerous complete bacterial genome sequences [1]. The detection of relatively recently acquired genes is possible because the laterally transferred genes have compositional features that distinguish them from vertically inherited genes [2]. However, finding a significant proportion of transferred genes in a bacterial genome does not mean that the entire gene transfer process that leads to the stable inheritance of new genes occurs frequently in the environment. Actually, the frequency of gene transfer between phylogenetically remote bacteria is expected to be low due to the requirement for several successive and rare events including colonization of the same environmental niche by donor and recipient bacteria, physical contact, compatibility for conjugation and transduction or DNA persistence and competence development when DNA is directly taken up by natural transformation [3]. Foreign DNA that has successfully penetrated a bacterial cell is integrated into the host genome by illegitimate recombination only if it escapes degradation by the restriction-modification systems (RM) [4] and the methyl-mismatch repair (MMR) system [5]. Due to these successive requirements, HGT with foreign DNA is unpredictable yet a single event occurring even once during bacterial evolution could fix a new trait in a bacterial lineage if the overall fitness is increased.

When donor DNA originates from an organism closely related to the recipient, recombination between similar or partially divergent (called homeologous [6]) sequences is much more likely. These transfer events will ensure genetic coherence and slow diversification when occurring within a group of closely related bacteria and will also promote environmental adaptation by sharing point mutations or transposon- and IS- mediated genetic rearrangements among the bacterial population [7]. However, bioinformatics methods cannot easily detect this new genetic information (unless significant numbers of individuals from the same species were completely sequenced) due to a lack of compositional features differentiating the donated DNA and the recipient genome [8]. In this case, the frequency of DNA transfer, the differences in transfer potential of different genes, and the potential impact on population fitness can be addressed by experimental approaches.

**Figure 1:**
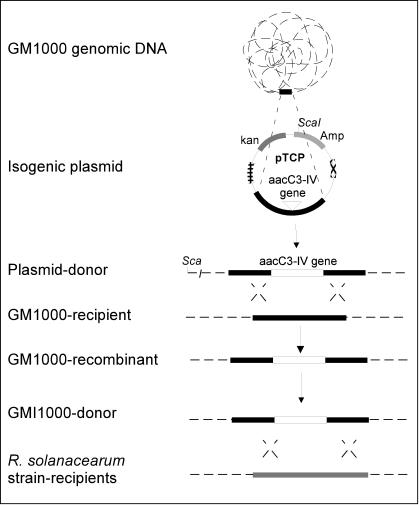
Experimental design to measure recombination rate at different positions of the *R. solanacearum* chromosome. Target positions on the chromosome (TCP) and on the megaplasmid (TMP) were identified and amplified by PCR. PCR products from the different genomic positions were cloned in appropriate vectors and afterwards, labeled with the *aacC3-IV* gene (gentamycin cassette). The pTCP (versus pTMP) plasmid carrying homologous GMI1000 fragments were linearized and resulting plasmids were used as donor to transform naturally the wild type strain GM1000 and recombination rate of each position designed. Total genomic DNA from *R. solanacearum* transformants and carrying *aacC3-IV* cassette resulting from double crossing-over were used as exogenous DNA donor to “re-transform” the wild type strain GM1000 and the CFBP2968, NCPPB332 and CFBP2957 strains to determine the recombination rate of genomic DNA. (Amp, ampicillin and Kn, kanamycin).

In this paper, we combined *in silico* and experimental approaches to study differences between these two types of HGT in bacteria. We used *R. solanacearum* as a model because of the evidence indicating a fundamental role of HGT in this plant pathogen's evolution. More than 7% of the genome was found to be encompassed by regions in which codon usage differed significantly from codon usage in the rest of the genome. In addition, most of these regions exhibited a base composition differing significantly from the G+C content for the entire genome indicating a foreign origin for these sequences [9]. Other authors using the Bayesian method estimated that about 16% of the genome was acquired by HGT [1]. Our first goal was to use alternate methods, including phylogenetic reconstruction of prokaryote homologous gene families and calculation of two codon usage indices to complete the list of genes acquired by HGT. These methods would be less biased than intrinsic codon usage-based approaches, which tend to overestimate the number of transferred genes, and in addition would identify putative donor microorganisms. These *in silico* studies were complemented by natural transformation experiments (Figure 1) in order to determine if and how this bacterium regulates acquisition of the DNA, including the genes detected as recently acquired by the bioinformatics analyses, that originates from the same or very closely related strains. *R. solanacearum* seems particularly appropriate for addressing these evolutionary questions because it is a naturally transformable bacterium whose cells use natural transformation to exchange genes at significant frequencies under *in planta* conditions [10]. Our hypothesis is that *R. solanacearum* bacterial cells are subjected to a constant flux of more or less homologous DNA in the open environment and that this flux might have led to the adaptive use of (“spandrel”) of DNA acquisition regulation mechanisms in order to generate genetic diversity without losing too much genomic stability. The term “spandrel” refers to the adaptive use of a function selected for another purpose [11], [12].

## Results

### Detection and bacterial origin of foreign genes acquired by *R. solanacearum* strain GMI1000

**Figure 2:**
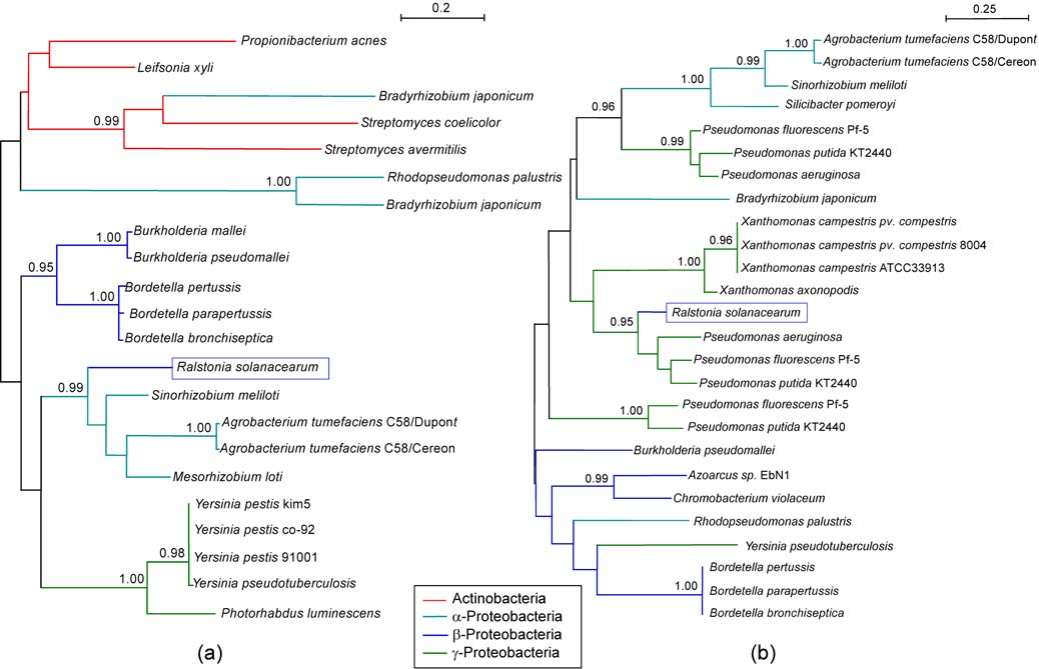
Two examples of HGT detected on the megaplasmid (a) and on the chromosome (b) through a phylogenetic approach. The two trees have been built respectively with the sequences from the HOGENOM families HBG007143, corresponding to the Proline imminopeptidase (a), and HBG225336, corresponding to the transcriptional regulator of Acetoin catabolism (b). The value for the Shimodaira-Hasagawa likelihood ratio test is given for the internal branches (only when *P*≥0.95). In both cases, the sequence from *R. solanacearum* is not clustered with the other β-proteobacterial sequences, this with a significant value for the clustering test.

In order to identify HGT events in the available *R. solanacearum* genome sequence, we used a phylogenetic approach to identify the putative donors of the newly acquired genes. With this approach, when *R. solanacearum* genes were not clustered with the other β-proteobacteria genes found in the family, and when this was supported by high probabilities for the Shimodaira-Hasagawa test, then we considered the possibility of an HGT event (Figure 2). When only a single β-proteobacteria other than *R. solanacearum* was present in a family, and when this bacteria was not clustered with *R. solanacearum*, we choose to ignore this family for the HGT count, since it was not possible to determine in which organism the transfer took place. We preferred this approach because it is less biased than codon usage-based methods, which tend to overestimate the number of transferred genes [13].

Using the phylogenetic trees, we found 151 inconsistencies in the 1139 phylogenetic trees examined, which represents 13.3% of the total families studied (the set of corresponding alignments and trees can be downloaded at ftp://pbil.univ-lyon1.fr/pub/datasets/PLoS07). However, because horizontally transferred genes tend to be orphans enriched in A+T nucleotides [14]–[16], we added to our list of putatively transferred genes those for which: (i) no available homologs were detected (and therefore no phylogenetic tree could be computed) or (ii) an extremely biased codon usage was found.

To do this, we computed the G+C content of the codon third position (G+C3%) and Codon Adaptation Index (CAI) [17]. The CAI reference table was built with all the genes coding for ribosomal proteins in *R. solanacearum*. After computing G+C3% and CAI values, we selected the 10% of the genes having the lowest values for these indices. This left us with 268 genes from the 3.7 Mb chromosome and 138 genes from the second replicon, the 2.1 Mb megaplasmid. Then, we performed a BLASTP search using these genes as templates in order to find those without homologs outside *R. solanacearum*. For that purpose, we set the E-value threshold at 0.1. We found a total of 42 genes from the chromosome and 37 genes from the megaplasmid matching these criteria. These orphan genes were also added to the list of putatively transferred genes. Together, phylogenetic reconstitution and codon usage selected about 15% of the genes in *R. solanacearum* genome that could be associated with HGT events and identified the potential bacterial donors.

All alignments and trees used to detect the transfers, as well as the list of orphans with high codon usage bias can be downloaded from the PBIL web server at ftp://pbil.univ-lyon1.fr/pub/datasets/PloS07.

### Selection of DNA positions for natural transformation experiments

Eighteen DNA positions on the genome encompassing a wide range of properties were selected to be amplified and cloned (Table 1). Fifteen of these positions were located on the chromosome (prefix TCP) and three positions were located on the megaplasmid (prefix TMP). Five DNA fragments (TCP4, TCP8, TCP14, TCP15 and TMP1) encompassed the DNA positions that were identified as being recently acquired by horizontal gene transfer by the phylogenetic reconstruction method (Table 1) and three DNA positions (TCP5, TCP14 and TMP2) contain DNA segments in which codon usage differed significantly from codon usage in the rest of the genome [9]. The TCP4, TCP8, TCP14, TCP15 and TMP1 positions carried RSc3437 (*vsr*), RSc0558 (*pilA*), RSc1815, RSc3252 and RSp0313 (*mexC*) genes respectively. The *vsr* gene (HOGENOM family HBG327419) was among the genes predicted by our phylogenetic approach as having been horizontally transferred and exhibits a strong codon usage bias toward A+T. Three others selected genes (RSp0313 (*mexC*), RSc0558 (*pilA*) and RSc1815 were apparently acquired from the γ-proteobacteria, *Acinetobacter baumannii*, *Pseudomonas aeruginosa* and *Xanthomonas campestris*, respectively. The RSc3252 gene was acquired from the least related organism, *Chlorobium tepidum*, which belongs to green sulfurous bacteria.

**Table 1:** Origin, properties of *Ralstonia solanacearum* (GM1000) genes used in this study as plasmid or genomic donor DNA; plasmid transformation frequencies and number of Chi-like sequences detected within the 2 kb long DNA positions.

Accession number	Gene location	Gene Acronym	Gene function	Putative Origin	Plasmid transformation frequencies	Number of Chi like 5′ cGCCGAAc 3′ within 2 Kb DNA fragment	Acronym for the targeted genomic positions^a^ (TCP^b^ versus TMP)
RSc3437	3710105	*vsr*	Avirulence	*Caulobacter crescentus (*α-proteobacteria)	1.29+/−0.20×10^−6^	1	TCP15
RSc3252	3506740	-	-	*Chlorobium .tepidum* (green sulfurous bacteria)	2.40+/−0.15×10^−6^	3	TCP14
RSc1815	1981107	-	-	*Xanthomonas campestris* (γ-proteobacteria)	2.60+/−0.09×10^−6^	3	TCP8
RSc0558	602739	*pilA*	Virulence	*Pseudomonas aeruginosa (*γ-proteobacteria)	1.14+/−0.26×10^−6^	1	TCP4
RSp0313	411136	*mexC*	-	*Acinetobacter baumannii* (γ-proteobacteria)	1.38+/−0.44×10^−7^	0	TMP1
RSc0828	869969	*tIS14b*	IS	Element of external origin	6.08+/−1.66×10^−6^	3	TCP5
RSc1921	2100591	-	Phage	Element of external origin	3.59+/−0.34×10^−6^	1	TCP9
RSc2585	2791556	*tn*	Transposon	Element of external origin	8.27+/−1.03×10^−6^	1	TCP12
RSc0458	489163	*ubiE*	House keeping	*R. solanacearum (*β-proteobacteria)	4.99+/−2.47×10^−8^	0	TCP2
RSc2191	2373410	*purD*	House keeping	*R. solanacearum (*β-proteobacteria)	4.12+/−1.43×10^−6^	1	TCP10
RSc2341	2538801	*ftsK*	House keeping	*R. solanacearum (*β-proteobacteria)	5.27+/−0.14×10^−7^	5	TCP11
RSc3023	3244061	*rpsG*	House keeping	*R. solanacearum (*β-proteobacteria)	1.40+/−0.29×10^−7^	3	TCP13
RSc0551	596177	*recA*	House keeping	*R. solanacearum (*β-proteobacteria)	1.66+/−0.26×10^−5^	6	TCP3
RSc1120	1176593	*comA*	House keeping	*R. solanacearum (*β-proteobacteria)	6.27+/−0.91×10^−7^	4	TCP6
RSc1151	1207216	*mutS*	House keeping	*R. solanacearum (*β-proteobacteria)	1.00+/−0.25×10^−5^	3	TCP7
RSc0171	192394	-	Putative gene	Putative gene	1.45+/−0.28×10^−6^	1	TCP1
RSp1328	1678048	-	Putative gene	Duplication	2.22+/−0.66×10^−6^	3	TMP2
RSp0877		*popA*	Virulence	*R. solanacearum (*β-proteobacteria)	nd	nd	TMP3

a TOPO recombinant plasmids with *R. solanacearum* inserts labelled by an *aacC3-IV* gene-cassette conferring resistance to gentamycin (Gm^R^)

b Targeted Chromosomal Position (versus Megaplasmid)

nd, not determined

Four DNA positions (TCP2, TCP10, TCP11 and TCP13) were selected because they contain well conserved house keeping genes (*ubiE*, *purD*, *ftsK* and *rpsG*). In addition, the megaplasmid TMP2 position was part of a 31 kb tandem position and TMP3 position encompasses a gene (*popA*) involved in plant pathogenicity. Four positions (TCP5, TCP9 TCP12 and TCP1) harbored an IS, a temperate bacteriophage, a transposon (TN) and a putative gene. Finally, DNA fragments, TCP3, TCP6 and TCP7, encompass genes whose functions are more or less directly involved in controlling genomic integrity (*recA, mutS*) and RSc1120, defined as coding for a DNA translocation protein which might be necessary for competence development (*comA-*like). A 2 kb DNA fragment from each of the 18 selected positions had the *aacC3-IV* gene, which confers resistance to gentamycin, inserted in its middle. The resulting recombinant plasmids were used as donor DNA in *R. solanacearum* natural transformation experiments (Figure 1).

### Transformation of *R. solanacearum* strain GMI1000 with plasmid-borne DNA fragments

**Figure 3:**
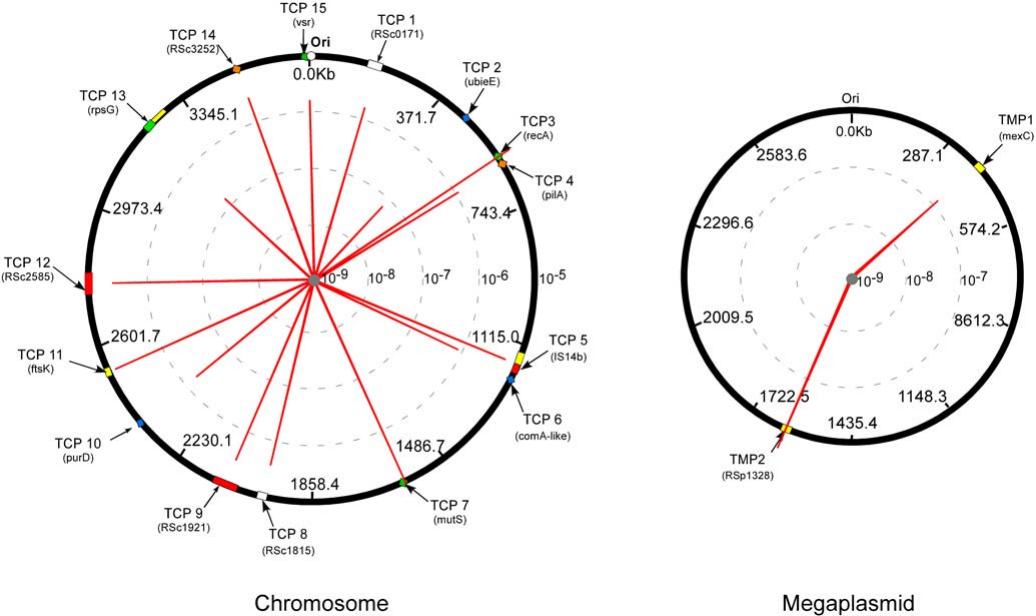
Recombination frequency variation between 17 *R. solanacearum* chromosome and megaplasmid borne DNA positions. Fifteen target positions on the chromosome (TCP) and three on the megaplasmid (TMP) were identified based on gene acquisition and function as described in the text. Plasmid donors were built following the general experimental design of figure 1. Recombination rate of these different genomic positions (proportional to red line) were measured after natural transformation and based on insertion of the *aacC3-IV* cassette by double crossing-over.

The TOPO plasmid vector without an insert did not produce any Km resistant recombinant *R. solanacearum* clones. Therefore, this plasmid was unable to replicate autonomously in *R. Solanacearum* GMI1000 strain and the integration frequency by illegitimate recombination was below the detection level. When plasmids contained inserts, DNA transformation frequencies varied by more than two orders of magnitude depending on the target position (Figure 3). The growth rate of transformants was not significantly modified in comparison to the wild type strain indicating that the differences in calculated transformation frequencies were not affected by differences in cell growth or survival (results not shown). The lowest frequency, which was found for pTCP2 (4.99+/−2.47×10^−8^), was 300 times lower than that of the highest frequency measured (pTCP3 at 1.66+/−0.26×10^−5^). Plasmids pTCP13 and pTMP1 also exhibited relatively low transformation frequency (1.40+/−0.29×10^−7^ and 1.38+/−0.44×10^−7^, respectively; Figure 3) although they were still 3 times higher than that for pTCP2. Several plasmids transformed *R. solanacearum* strain GMI1000 at frequencies between one and two orders of magnitude higher than pTCP2. These included pTCP1, pTCP4, pTCP6, pTCP8, pTCP9, pTCP10, pTCP12, pTCP14, pTCP15 and pTMP2. Finally, pTCP5, pTCP7, pTCP11 and pTCP3 yielded transformants at frequencies more than two orders of magnitude higher than pTCP2. All the gentamycin resistant *R. solanacearum* clones tested resulted from double crossing over events according to their sensitivity to kanamycin and the size of the PCR products, which were all about 4 kb long as expected for gene replacement (results not shown).

### Transformation of different *R. solanacearum* strains with linear chromosomal DNA fragments from *R. solanacearum* strain GMI1000

The total genomic DNA (composed of fragments ranging in size between 40 and 100 kb) from the different plasmid-transformed *R. solanacearum* GMI1000 clones was used as donor DNA with GMI1000, CFBP2968, NCPPB332 and CFBP2957 as recipient strains. Conditions for homologous recombination were established when GMI1000 strain was the recipient strain as the only difference between donor DNA from the same strain and the recipient genome was the marker gene inserted in the targeted DNA position. Recombination conditions for other recipient strains varied from homeologous to heterologous as strains CFBP2968 (phylotype I), NCPPB332 (phylotype III) and CFBP2957 (phylotype II) have respectively ∼98%, ∼81% and ∼69% of the GMI1000 genes conserved in their genomes [18]. In addition, these three strains were the most efficiently transformed among all strains tested in each phylotype and PCR carried out with GMI1000 primers for six out of the 18 positions tested confirmed presence of the corresponding genes in these isolates (results not shown).

**Figure 4:**
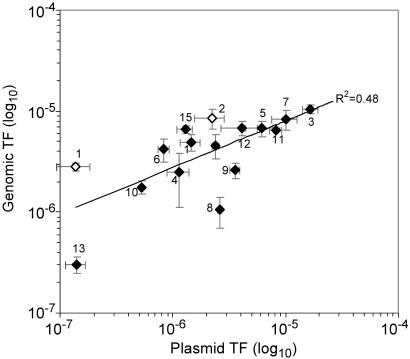
Correlation between recombination frequencies obtained with plasmid DNA and genomic DNA after natural transformation of *R. solanacearum* GMI1000 as recipient. Donor DNA belongs to the same strain and was either plasmid DNA containing 2 kb long *R. solanacearum* DNA or genomic DNA from recombinant strains (see the text). The numbers along the curves refer to the DNA position acronym with white and black symbols for chromosome and megaplasmid positions, respectively (TCP2<detection limit, TMP3, not determined). TF, transformation frequency.

Again, significant differences in transformation frequencies were detected for different genomic positions. In general, the DNA positions for the strain GMI1000 that exhibited the lowest transformation frequencies when recombination was mediated by plasmid borne DNA fragments also yielded low transformation rates when genomic DNA was used (Figure 4 and Table S1). This included gTCP1 and gTCP13 with recombination frequencies<10^−10^ and 3+/−0.06×10^−7^, respectively (Figure 4 and Table S1). Similarly, the highest transformation frequencies were observed when recombination was mediated by DNA positions (e.g., gTCP3 and gTCP7), which also yielded the highest frequency when they were located on a plasmid. However, comparison of transfer frequency between plasmids and genomic DNA of the same strain (GMI1000) for the other targeted DNA positions showed some discrepancies in spite of a significant (p<0.01) correlation between the two experiments (correlation coefficient = 0.69; Table 2).

**Table 2:** Correlation coefficients of plasmid DNA recombination frequencies with the genomic DNA recombination frequencies and with the main physico-chemical parameters of the seventeen 2 kb long DNA fragments targeted in this study.

	Recombination frequencies of genomic DNA	Distance of Origin	GC%	GC skew	DNA helical stability
Pearson correlation	0.691^a^	0.031	0.021	0.062	−0.024
*P*-values	0.003	0.890	0.936	0.813	0.926

a indicated that the correlation is significant (p<0.01).

**Figure 5:**
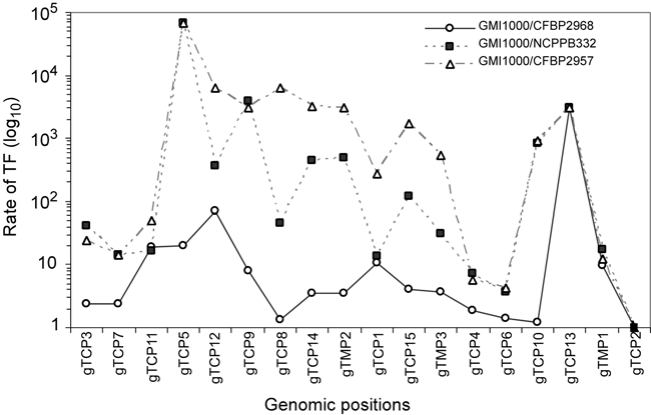
Ratio of recombination frequencies after natural transformation of different distantly *R. solanacearum* strains. Total genomic DNA of GMI1000 strain was used as donor with GMI1000, CFBP2968, NCPPB332 and CFBP2957 as recipient strains. These strains CFBP2968, NCPPB332 and CFBP2957 have respectively 98%, 81% and 69% of the GMI1000 genes conserved in their genomes (TCP2<detection limit). TF, transformation frequency.

With the other *R. solanacearum* strains as recipients (Figure 5), the transformation frequencies were systematically lower than with GMI1000 whatever the DNA positions tested and higher genomic divergence between the recipient strain and GMI1000 correlated with greater decreases in transformation frequencies (Figure 5). Moreover, the range between the highest and the lowest transformation frequencies also increased with overall genome divergence reaching more than 4 orders of magnitude for strains NCPPB332 and CFBP2957 while it was less than two orders of magnitude for the two most closely related strains (GMI1000 and CFBP2968).

In spite of the increasing range of transformation frequencies with increasing genomic divergence, the DNA positions that yielded the highest transformation frequencies with GMI1000 (TCP3, TCP6, and TCP7) systematically yielded the highest frequencies for each recipient (Figure 5). The lowest transformation values observed for these strains corresponded to DNA positions that also yielded the lowest frequencies when recombination was homologous. In addition, the high frequency regions were less susceptible to increasing genomic divergence.

### Role of Chi*-*like sequences

Physico-chemical parameters of the seventeen 2 kb long DNA fragments targeted in this study were calculated. These parameters, including GC%, GC “skew”, genome (chromosomal or plasmid) localization, distance from the origin at replication and the denaturing free energy (melting point), did not significantly correlate to recombination frequencies (Table 2).

**Figure 6:**
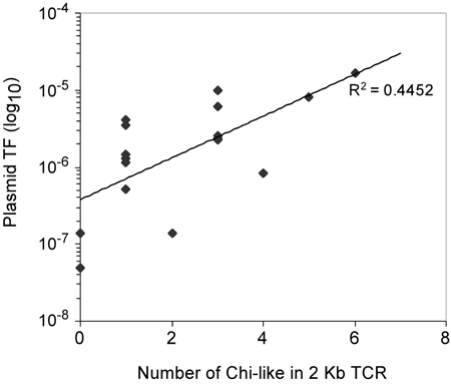
Correlation between plasmid recombination frequencies and the number of Chi-like motifs within the 2 kb long DNA fragment. TF, transformation frequency.

The 2 kb DNA sequences were examined in order to detect the presence of the longest repeated motifs, which might significantly correlate to the recombination frequencies: for each length = L with L being 7 and 8, we counted the occurrences of all the words of length L, and for each word we measured the correlation of the ranks between these numbers of occurrences and the frequencies, using a Spearman test. For L = 7, the two best words were 5′ cGCCGAA 3′ (p-value = 10^−3.73^) and 5′ GCCGAAc 3′ (p-value = 10^−3.08^) and for L = 8 the best word was 5′ cGCCGAAc 3′ (p-value = 10^−3.06^). These motifs were detected in 15 of the 17 fragments with 9 of them containing at least 3 copies of these specific sequences (Table 1). The number of motifs occurring in a given region correlated (R^2^ = 0.48) to recombination frequencies (Figure 6). The three DNA positions that exhibited the highest transformation frequencies (TCP3, TCP7 and TCP11) contained two copies each of the consensus sequence (Table 1). The genes that yielded the lowest transformation values including *ubiE* (TCP2), and *mexC* (TMP1), did not contain any sequence inside the position associated with the donor plasmids (Table 1).

A whole genome analysis detected that the consensus DNA sequence, 5′ GCCGAA 3′, with a “c” located at the beginning and/or the end of the word was present on the *R. solanacearum* genome at a high frequency (average 2080+/−67 fold for the two best words with 7 letters and 751 fold for the consensus word). To test the uniformity of the repartition of the two consensus sequences, we used the classical Watson test of goodness of fit [19]. We found that the distribution of the 5′ GCCGAAc 3′ motif is non-uniform on both strands of the chromosome while the distribution of the 5′ cGCCGAA 3′ motif is uniform on the leading strand and non-uniform on the lagging strand. For the megaplasmid, on the other hand, distribution of both motifs was found to be uniform (*i.e.*, random) on the two strands (Table 3).

**Table 3:** Results of the Watson uniformity test for the two Chi motifs on the *R. solanacearum* chromosome and megaplasmid.

Replicon	Motif Chi-like	Strand	*P*-value^a^
Chromosome	5′ cGCCGAA 3′	+	*P*<0.01
		−	*P*<0.01
	5′ GCCGAAc 3′	+	0.05<*P*<0.10 (ns)
		−	*P*<0.01
Megaplasmid	5′ cGCCGAA 3′	+	*P*>0.10 (ns)
		−	*P*>0.10 (ns)
	5′ GCCGAAc 3′	+	0.05<*P*<0.10 (ns)
		−	0.05<*P*<0.10 (ns)

a *P*-values are given for the different combinations of motifs and strands. All *P*-values>0.05 are considered non-significant (ns).

These motifs could be Chi-like sequences with a putative role similar to that found for Chi sequences in *E. coli* for the attenuation of RecBCD exonuclease activity and the promotion of RecABCD-mediated homologous recombination [20].

## Discussion

### Detection of laterally transferred genes in the *R. solanacearum* GMI1000 genome sequence

Analysis of the complete sequence of the *R. solanacearum* strain GMI1000 with the phylogenetic tools presented earlier confirms that this bacterium has acquired numerous genes from other micro-organisms. For example, the *vsr*-like gene was possibly acquired from a α*-proteobacteria*, *Caulobacter crescentus* providing *R. solanacearum* with a mechanism for repairing mismatches in addition to MMR even if, in the case of *vsr*, the repair is limited to very short patches of DNA [21]. In *E. coli*, MMR and VSP have complementary effects with efficiencies varying during the bacterial life cycle [22]. If *R. solanacearum* behaves similarly, then MMR could be expressed mainly when the pathogen multiplies actively in plant tissues and VSP during its less active life in the soil. If VSP contributes to increased genome stability under stringent environmental conditions, the overall fitness of the bacterium would be improved, thus, justifying the fixation of the *vsr* gene in the population.

The bio-informatics analysis also detected the putative acquisition of *pilA* and *mexC* from *Acinetobacter baumannii* and *Pseudomonas aeruginosa,* respectively. Both belong to the γ-class of proteobacteria. These genes, *pilA* and *mexC*, encode for cell envelope components and are involved in the formation of type IV fimbrial pilin signal peptide proteins and efflux pump antibiotic resistance proteins, respectively [23], [24]. Apparently, these new genes were sufficiently beneficial to *R. solanacearum* that they were fixed in its genome. Examples of other potentially transferred genes are RSc3252 and RSc1815 that originated from *Chlorobium tepidum* (green sulfur bacteria) and *Xanthomonas campestris* (γ-proteobacteria), respectively, but these genes have only putative functions. However, all genes detected as recently acquired are not necessarily beneficial to their new hosts, because either their presence resulted from co-transfer events and/or the selection process has not yet deleted the unnecessary DNA fragments. The half-life of unnecessary DNA in bacterial genomes is unknown.

### *R. solanacearum* as a realistic and useful model to study intra and inter-species crosses

The first requirement for HGT to occur is the contact between donor cells or their DNA and the recipient bacteria. In addition to soil and rhizosphere environments, the plant pathogen *R. solanacearum* colonizes numerous plants [25] leading to the development of opportunistic soil bacteria in the degraded plant tissues [26]. The probability of transformation by these foreign genes is increased further by the competence development of *R. solanacearum in planta* as demonstrated by greenhouse experiments [10]. The ecology of different potential donor bacteria including *Xanthomonas campestris*, which is also a pathogen for plants, is compatible with that of *R. solanacearum*. The only marked exception would be the strictly anaerobic and obligate autotrophic *C. tepidum*, which is typically found in anoxic and sulfide-rich waters, mud, sediments, and microbial mats [27]. Other bacteria including human pathogenic bacteria such as *A. baumannii* and *P. aeruginosa*, can live transiently in soils [28] and the *alpha*-purple aquatic bacterium *C. crescentus* is found in all types of water, including lakes, streams, sea water and waste water [29], [30]. The possibility that most of these bacteria colonize alternate habitats, such as plant tissues, cannot be excluded although this was never reported.

Under *in planta* conditions as evidenced in experiments under greenhouse [26], competent *R. solanacearum* cells are subjected to a flux of exogenous DNA, including plant and opportunistic bacterial DNA and its own DNA released by dying cells. Foreign DNA is degraded by the MMR and other systems except in the rare cases when illegitimate recombination mediates their integration into the genome. The fate of *R. solanacearum* DNA is totally different, inoculation experiments involving two *R. solanacearum* strains demonstrate that natural transformation mediated gene transfer occurs at high frequency under *in planta* conditions [10]. Sequence similarity between the incoming DNA and the recipient genome leads to integration of *R. solanacearum* genes by homologous (or homeologous in the case of more than one infecting strain) recombination at frequencies several orders of magnitude higher than for heterologous DNA. This combination of ecological, physiological and genetic conditions including extensive clonal multiplication, natural release of DNA, competence development, DNA uptake and genome integration, demonstrates the interest in *R. solanacearum* as a model for studying HGT regulation.

### Homologous recombination as the critical step that regulates gene acquisition

Our hypothesis was that bacteria might regulate acquisition of homologous genetic material so that some genes accumulate genetic diversity while other genes maintain a higher stability level. The strategy used here was to test the frequency of homologous and homeologous recombination-mediated integration of a marker gene cloned into different positions of the *R. solanacearum* GMI1000 genome by using as recipients the same strain, GMI1000 and three other strains, CFBP2968, NCPPB332 and CFBP2957, that exhibited an overall genomic divergence relative to GMI1000 of 2, 19 and 31%, respectively [18]. These *in vitro* transformation tests simulated the *in planta* situation where donors and recipients resulted from the clonal multiplication of bacterial cells belonging to one or more *R. solanacearum* strains.

Our results demonstrated that DNA fragments from various genomic positions of the same strain transformed the recipient strains at frequencies markedly different even under totally homologous DNA conditions. The *recA-* and *mutS-* gene containing positions were identified as natural transformation and certainly homologous recombination “hot spots” (see below). Previous studies suggest that the uptake of DNA by *R. solanacearum* would not require the presence of specific sequences to bind the cell wall for the donor DNA to be processed into the cell [31]. Although regulation of this uptake stage cannot be totally excluded, transformation frequency differences would more likely result from differences of recombination efficiency between the different DNA fragments. In addition, DNA uptake control might be linked in part to nutrient requirements [32]. Whether the mechanism associated with DNA uptake defines the fate of the DNA once introduced into the cell is less clear, but the possibility that DNA uptake began as a nutrient uptake mechanism cannot be discounted [32]. A recent bioinformatics study suggest that at least for some classes of short DNA sequences, DNA uptake is biased by sequence definition, which is not necessarily consistent with nutrient driven uptake [33].

Transformation-recombination frequency decreased with sequence divergence for the three divergent strains, but the decrease was far from being identical for the various DNA positions tested. Interestingly, the DNA positions that were classified as “hot spots” with GMI1000 as recipient were also those for which transformation-recombination frequencies remained the highest and changed the least. Even with the least related strain (CFBP2957), transformation frequencies at the “hot spots” were only one order of magnitude lower than those for the homologous GMI1000, while frequencies decreased by 4 orders of magnitude for the DNA positions that transformed all strains at the lowest frequency.

Frequency differences are not apparently related to variable sizes of donor DNA fragments since transformation tests carried out with the plasmids (2kb in size) provided results consistent with those obtained with chromosomal fragments. PCR primers were designed to amplify a 2 kb long DNA fragment that was subsequently cloned into the plasmids. The resulting 1 kb long DNA fragments that flanked both sides of the marker gene were significantly longer than the minimal length necessary for efficient homologous recombination. Moreover, there was no theoretical limitation in the length of DNA fragments when chromosomal DNA with the marker gene inserted in the targeted position was used to transform the wild strain. Frequency variation could not be related to DNA physical or chemical parameters either. These results would indicate that differences in transfer frequency are only related to the nucleotide sequence of the DNA positions on which homologous recombination occurs. This would suggest that the genes present in a bacterial genome do not exhibit the same *sensu stricto* potential to be transferred even into a new isogenic host.

### Involvement of Chi-like sequences

The sequences in the targeted genomic positions were analyzed and Chi-like (‘5-cGCCGAAc-3’) sequences were detected that might explain differences in homologous and homeologous recombination frequencies. The experimental results obtained with the four recipient strains were in general agreement, and thus, strengthened the hypothesis for the involvement of Chi-like sequences during recombination initiation. The highest transfer frequencies were found for fragments that contained more than two Chi-like sequences, thus, indicating that accumulation of these sequences could create “hot spots” for homologous recombination. Our results indicate that the Chi-like sequences are not distributed randomly in the *R. solanacearum* chromosome confirming what was already reported in other bacteria (e. g. *E. coli*, *Bacillus subtilis*, *Haemophilus influenzae* and *Lactococcus lactis)* [34]. However, distribution of these motifs was found to be uniform on the *R. solanacearum* megaplasmid, a surprising (and unexplained) result that could be related to the involvement of replication mechanisms that differ between the 2 replicons.

The interest in using recombinant plasmid-borne fragments as donor DNA was that the sequence analysis was restricted to the 1 kb long DNA fragments flanking the marker gene eliminating the possible influence of the Chi-like sequences located further upstream of the targeted fragments even if, on the other hand, the use of the entire genome extracted from recombinant strains was ecologically more realistic. Therefore, the reduced differences found between the lowest and the highest transformation frequencies with genomic borne fragments could be explained by the involvement of Chi-like sequences at some unknown distance upstream.

In *E. coli,* Chi sequences are recombinational hotspots at which enzymes bind preferentially to repair DNA damaged by ionizing radiations or by the collapse of a replication fork when passing single-strand nicks [35]. The ends of the broken DNA on double strands are processed by the multi-functional enzyme complex RecBCD involving successively a helicase activity to split the duplex into its component strands and a nuclease activity to digest them. At a Chi site, the nuclease activity is attenuated and the RecBCD loads RecA onto the 3′ tail of the DNA to initiate recombination. The foreign DNA acquired by HGT could be perceived by recipient cell as damaged DNA and be processed by the same enzymes [36] with necessarily a critical role for Chi sequences as preferential sites to initiate recombination. Our results indicate that, in addition to a putative implication in the repair of endogenous damaged DNA like in *E. coli*, the Chi-like sequences in *R. solanacearum* (which possesses *addAB* genes having the same functions as *recBCD* in *E. coli*) could be key components of the adaptation potential by permitting the cell to regulate the gene acquisition process as already proposed in other naturally transformable bacteria such as *B. subtilis* and *H. influenzae* [34].

Chi-like sequences strongly limit the influence of sequence divergence, which usually decreases recombination efficiency dramatically [36], [37]. For instance, our results demonstrate that DNA exchange frequency for some DNA positions remains very high in spite of a significant overall genomic divergence between strains GMI1000 and CFBP2957 (up to 30%), a level that led to classifying these strains as two separate genomic species [38]. These results feed the debate on species boundaries in bacteria, on the strength of biological barriers to regulate DNA exchange, and confirm the difficulty to adapt a bacterial species concept that would be based on genomic coherence between members of a same species sharing an exclusive common gene pool [39].

### Transformation “hot spots” in *R. solanacearum* and recombination potential of mobile elements and recently acquired genes

Two of the main transformation “hot spots” detected in this study were the genomic positions (TCP3 and TCP7) containing the *recA* and *mutS* genes, which are involved in DNA repair and recombination. This could be justified by the need to maintain stability and integrity in DNA positions containing important housekeeping genes [7]. By analogy to *E. coli*, damaged endogenous DNA reparation efficiency is certainly increased by presence of Chi-like sequences to initiate recombination. However, genomic stability of these positions could also benefit from their spread at high frequency among bacteria that reduces the risk of genetic drift by point mutations in separate lineages. In addition, a recombination “hot spot” in the *mutS* gene is in agreement with the hypothesis involving HGT as a mechanism for *mutS* negative mutators to re-acquire a functional *mutS* copy to return to a more stable wild type phenotype [40], [41].

Surprisingly, the genomic positions containing mobile elements, such as insertion sequences, prophages and transposons that have developed specific mechanisms to displace from place to place within and among genomes exhibited a transformation potential significantly lower than the hot spots that carry *mutS* and *recA* genes. Our study also included genomic positions with genes acquired from other phyletically remote bacteria by HGT. These positions did not exhibit any copy of the specific Chi-like sequences detected in the recombination “hot spots”. Moreover, transformation frequency of the fragment containing the *vsr* gene was respectively 13 and 8 times lower than for those containing *recA* or *mutS* genes indicating that the corresponding positions should not be considered as “hot spots” for transformation. These data would indicate that acquisition of foreign genes might not be regulated as could be *R. solanacearum* genes but would rather result from the combination of several events happening mainly by chance and at very low frequency including the uptake of exogenous DNA and its integration and/or by rearrangement in the genome post-HGT.

### The two sides of HGT in bacteria

According to bioinformatics analysis of genome sequences (this study) and inoculation experiments in plant tissues [10] *R. solanacearum* like other bacteria seems to use two complementary HGT-based strategies to optimize adaptation. The first one is based on acquisition at high frequency of DNA from more or less closely related cells. This type of HGT would permit on one hand to maintain stability and integrity in some important DNA positions, a constant DNA homogenization reducing the risk of genetic drift [7], and on another hand to spread potentially beneficial mutations efficiently among the population. However, our data demonstrate that the various DNA positions are transferred at significantly different frequencies indicating a possible regulation mechanism. As for other functions in bacteria, the fundamental evolutionary question is whether this property might have been specifically selected to increase adaptation potential or if it might be the side effect of cellular mechanisms in charge of DNA repair. Whatever the response (about the evolutionary process), bacteria in the open environment are confronted with genes that do not transfer at the same frequency and thus with evolutionary implications that cannot yet be precisely evaluated. The high frequency with which the preferentially transferred DNA positions are transformed in other strains indicates that such HGT events must have a strong impact on genome evolution and must significantly contribute to the adaptation potential of the bacteria.

The second adaptive strategy of bacteria is to acquire genes from unrelated bacteria. The *in silico* analyses of the *R. solanacearum* genome sequence detected genes that were laterally transferred from a wide range of remotely related bacteria. Their fixation suggests that they also contributed to increase the adaptation potential of their new host. However, our experimental transformation results do not indicate that these laterally acquired DNA positions belong to the transformation-recombination “hot spots” detected for other typical *R. solanacearum* genes. This might mean that their acquisition resulted from a hypothetical contact with a donor bacteria and a successful integration by an extremely rare illegitimate recombination event. The likelihood that this was directly related to the active Chi-like sequences based mechanism, which leads to some other genes to be exchanged at high frequency, seems to be low. However, successful transfer to genomic “hot spots” and subsequent mobility to more stable genomic positions through mobile elements such as insertion sequences cannot be disproved.

## Materials and Methods

### *Ralstonia solanacearum* strains

The four strains used in this study are classified in the *R. solanacearum* species complex and belong to phylotype I (GMI1000, CFBP2968), phylotype II (CFBP2957) and phylotype III (NCPPB332) [18]. These strains that exhibit sensitivity to ampicillin, kanamycin and gentamycin were cultured at 28°C in the complete B medium [42] and exhibit the same natural transformation frequency when transformed by their own DNA (results not shown).

### Phylogenetic bioinformatics analysis

For the phylogenetic inference, 2039 homologous gene families containing at least one sequence from *R. solanacearum* GMI1000 and from other β-proteobacteria were extracted from the HOGENOM database (http://pbil.univ-lyon1.fr/databases/hogenom.html). After excluding eukaryotic sequences, multiple alignments of the families were computed using MUSCLE [43], with all default parameters. These alignments were then filtered with GBLOCKS [44] in order to keep only their reliable parts. Using these filtered alignments, we kept only families containing a number of sites equal at least to 1.5× the number of taxa in the families. Only 1139 families remained after this final selection. Phylogenetic trees were computed (with 100 bootstrap replicates) on the remaining alignments with the fast Maximum-Likelihood method implemented in PHYML [45].

Under PhyML, the WAG amino acid substitution model [46] was used, and across-site rate variation was modelled by a Gamma distribution with four classes of substitution rates. Estimation of the Alpha parameter for Gamma distributions was carried out by PhyML. Trees were then manually checked to detect those in which *R. solanacearum* was not grouped with the other(s) β-proteobacteria, this with a Shimodaira-Hasegawa likelihood ratio test≥95% [47].

Phylogenetic trees computations were performed on the IN2P3 Linux cluster containing more than 1300 CPUs.

### Plasmid construction

Eighteen oligonucleotide pairs (Table S2) were designed according to the complete nucleotide sequence of *R. solanacearum* in order to amplify a 2 kb long DNA fragment for each selected position by PCR. PCR primers were designed by using the OLIGO 5.1 software (National Biosciences, Inc. NBI) applied to the complete GMI1000 sequence. The resulting PCR products were ligated to one of the following plasmids, pUC19, pBluecript or pCR® 2.1-TOPO vector or pGEMT®-T/Easy vector (InVitrogen France, Promega, France) depending on the restriction sites available for further restriction cleavage and cloned in *Escherichia coli* strain (DH5α) according to the manufacturer instructions. Recombinant plasmids were extracted and purified with the QIAprep Mini-prep Kit (Qiagen SA, Germany) and re-suspended in sterile purified water. Each plasmid was digested with the appropriate restriction enzyme (Table S3) that cut the insert once, approximately in the middle in order to clone the *aac*C3-IV gene conferring resistance to gentamycin (Gm^R^) [48]. The resulting plasmids were extracted and the construction was verified by electrophoresis on agarose gel after digestion with the appropriate restriction enzymes. PCR products that were initially cloned in the pGEM-T, pUC19 and pBluescript vectors were amplified again with the same initial primers and recombinant plasmids as template before the chimeric construction was cloned in the pCR® 2.1-TOPO vector. The 18 pCR® 2.1-TOPO-derivative resulting plasmids contained all the same vector background with the *bla* and *nptII* genes conferring resistance to ampicillin (Amp^R^) and kanamycin (Kn^R^) respectively and each contained a specific *R. solanacearum* 2 kb genomic DNA fragment in which the *aacC3-IV* gene conferring resistance to gentamycin (Gm^R^) had been inserted, approximately in the middle.

### Preparation of transforming DNA

The plasmids were extracted and purified by using the QIAprep Mini-prep Kit before they were digested with *ScaI*. The only exception was plasmid pTCP9 that was treated by *CpoI* because of presence of a *ScaI* site in the insert (Table S3). These 2 enzymes *ScaI* and *CpoI* cut only once in the *bla* gene and the *nptII* gene respectively, linearizing the plasmid from the vector background without affecting the *R. solanacearum* fragments and the marker gene. Restricted plasmid DNA was subsequently purified with the GFX™ PCR DNA and gel purification kits (Amersham Biosciences Germany) before used in transformation tests. *R. solanacearum* genomic DNA used in transformation was extracted from gentamycin resistant and kanamycin sensitive recombinant strains after they were transformed with the recombinant plasmid set presented above according to the available protocol [31].

### *R. solanacearum* natural transformation

Natural transformation of the *R. solanacearum* strains was carried out according to the procedure described by Bertolla et al. [31]. Briefly, cells of *R. solanacearum* were grown in minimal medium (MM) to an O.D. _600 nm_ = 0.8 (about 5×10^8^ cells ml^−1^). Fifty micro-liters of this cell suspension were incubated with either 100 ng of plasmid DNA or 400 ng of genomic DNA on polycarbonate membranes (Millipore, Ireland) deposited on the surface of solid MM (MMG) medium and incubated for 48 h at 30°C. Bacterial cells were then harvested from the membrane surface and suspended again in 5 ml of sterile water. A 500 µl aliquot was used to inoculate rich BG agar medium plates containing respectively gentamycin (12.5 µg ml^−1^) (Euromedex, France) in transformation tests with genomic or plasmid DNA or both gentamycin (12.5 µg ml^−1^) and kanamycin (25 µg ml^−1^) (Euromedex, France) in transformation tests with plasmid DNA. The recipient population was enumerated by plating appropriate dilutions on the BG medium without any antibiotics. Recombinant *R. solanacearum* colonies that exhibited resistance to gentamycin but sensitivity to kanamycin were those in which a double cross over event replaced the wild type gene by the chimeric construction while resistance to kanamycin indicated that the plasmid was totally integrated following a single cross over event (Figure 1). Integration of the cassette by single or double cross over events was verified by PCR for 3 randomly selected clones for each construction. Controls included transformation tests carried out without DNA that allowed determining spontaneous mutation frequency. Other control experiments used the plasmid DNA from non recombinant TOPO vector or the DNA from the wild type strain GMI1000 as transforming DNA. Each filter experiment was done at least in triplicate and all calculated transformation frequencies are given as the mean value. A statistical *t* student test was performed to evaluate significance of differing DNA source used.

### Detection of Chi –like sequences

The 2 kb DNA sequences corresponding to *R. solanacearum* DNA fragment cloned into plasmids were analyzed by using the R software (seqinR [49]) to detect the presence of the longest repeated motifs, which might significantly correlate to the recombination frequencies. The occurrence of the words in *R. solanacearum* whole genome was determined using the Fuzznuc online program (http://bioweb.pasteur.fr/docs/EMBOSS/fuzznuc.html) which belongs to the EMBOSS package.

## Supporting Information

Table S1 (0.08 MB DOC)

Table S2 (0.07 MB DOC)

Table S3 (0.08 MB DOC)
