# Supplementary material for: Horizontal Gene Transfer Regulation in Bacteria as a “Spandrel” of DNA Repair Mechanisms
Source: PLoS One. 2007 Oct 24;2(10):e1055. doi: 10.1371/journal.pone.0001055 (PMC2013936; doi:10.1371/journal.pone.0001055)
Supplement: Table S1 — (0.08 MB DOC) [file pone.0001055.s001.doc]

| *R. solanacearum* strain acronym | GM1000 | CFBP2968 | NCPPB332 | CFBP2957 |
| --- | --- | --- | --- | --- |
| Conserved genes (%) | 100 | 98 | 81 | 69 |
| gTCP1 | 5.0 x 10^-6^ | 4.8 x 10^-7^ | 3.6 x 10^-7^ | 1.9 x 10^-8^ |
| gTCP2 | <1.0 x 10^-10^ | <1.0 x 10^-10^ | <1.0 x 10^-10^ | <1.0 x 10^-10^ |
| gTCP3 | 1.0 x 10^-5^ | 4.2 x 10^-6^ | 2.4 x 10^-7^ | 4.0 x 10^-7^ |
| gTCP4 | 2.5 x 10^-6^ | 1.3 x 10^-6^ | 3.5 x 10^-7^ | 4.4 x 10^-7^ |
| gTCP5 | 6.8 x 10^-6^ | 3.3 x 10^-7^ | <1.0 x 10^-10^ | <1.0 x 10^-10^ |
| gTCP6 | 4.2 x 10^-6^ | 3.0 x 10^-6^ | 1.1 x 10^-6^ | 9.6 x 10^-7^ |
| gTCP7 | 8.4 x 10^-6^ | 3.5 x 10^-6^ | 5.9 x 10^-7^ | 6.0 x 10^-7^ |
| gTCP8 | 1.1 x 10^-6^ | 8.3 x 10^-7^ | 2.4 x 10^-8^ | 1.7 x 10^-10^ |
| gTCP9 | 2.6 x 10^-6^ | 3.2 x 10^-7^ | 6.7 x 10^-10^ | 8.3 x 10^-10^ |
| gTCP10 | 1.8 x 10^-6^ | 1.5 x 10^-6^ | 2.2 x 10^-9^ | 2.0 x 10^-9^ |
| gTCP11 | 6.6 x 10^-6^ | 3.4 x 10^-7^ | 3.9 x 10^-7^ | 1.3 x 10^-7^ |
| gTCP12 | 6.8 x 10^-6^ | 9.7 x 10^-8^ | 1.9 x 10^-8^ | 1.1 x 10^-9^ |
| gTCP13 | 3.0 x 10^-7^ | <1.0 x 10^-10^ | <1.0 x 10^-10^ | <1.0 x 10^-10^ |
| gTCP14 | 4.6 x 10^-6^ | 1.3 x 10^-6^ | <1.0 x 10^-8^ | <1.4 x 10^-9^ |
| gTCP15 | 6.6 x 10^-6^ | 1.6 x 10^-6^ | 5.6 x 10^-8^ | 3.8 x 10^-9^ |
| gTMP1 | 2.8 x 10^-6^ | 2.9 x 10^-7^ | 1.6 x 10^-7^ | 2.2 x 10^-7^ |
| gTMP2 | 8.5 x 10^-6^ | 2.4 x 10^-6^ | 1.8 x 10^-8^ | 2.8 x 10^-9^ |
| gTMP3 | 7.2 x 10^-6^ | 2.0 x 10^-6^ | 2.3 x 10^-7^ | 1.3 x 10^-8^ |

**Table S1.** Transformation-recombination frequencies obtained in four strains of *Ralstonia solanacearum* after natural transformation by genomic DNA extracted from *aacC3-IV* gene tagged GMI1000 derivatives.

Recombination conditions varied from homologous when strain GMI1000 was used as donor and recipient to homeologous and illegitimate for recipient strains CFBP2968, NCPPB332 and CFBP2957 that exhibited an increased taxonomic divergence rate to the donor DNA strain. Recombination frequencies presented here are the mean of three independent replicates.
