# Supplementary material for: Horizontal Gene Transfer Regulation in Bacteria as a “Spandrel” of DNA Repair Mechanisms
Source: PLoS One. 2007 Oct 24;2(10):e1055. doi: 10.1371/journal.pone.0001055 (PMC2013936; doi:10.1371/journal.pone.0001055)
Supplement: Table S2 — (0.07 MB DOC) [file pone.0001055.s002.doc]

**Table S2.** List of primers used to amplify the different *R. solanacearum* genomic DNA positions.

| PCR primer acronyms | Targeted position accession number (gene acronym) | Primer nucleotide sequence | Target localization^a^ |
| --- | --- | --- | --- |
| F2257U / F2258L | RSc0171 | ACCGCCCGCATAAGCAAT / CCCGTCCCGCTGTTCAA | Chr |
| F2112U / F2113L | RSc0458 (*ubiE*) | ACGCGCTGTGCTATCCC / GGCCGAACTTGGACAACA | Chr |
| F1022U / F1023L | RSc0551 (*recA*) | CTTGCGGCCAGTTTA / CGTACCGGCAAAGCA | Chr |
| F2092U / F2093L | RSc0558 (*pilA*) | CGCGTCGAACCCAAGAAC / GGCTTCCGCTCATCGTCA | Chr |
| F2114U / F2115L | RSc0828 (*tIS14b*) | TGCCAAATGTCGCTGCTG / TGCCCAACCGCCATACC | Chr |
| F1024U / F1025L | RSc1120 (*comA*) | CGAGCTGCCCGAAGT / CCTGCGGATGCGGAT | Chr |
| F530U / F1569L | RSc1151 (*mutS*) | GCGTGTGGCTGCGGTTGTGC / GACAAGCACACGCCGATGATG | Chr |
| F2253U / F2254L | RSc1815 | AGGCGCAACTGCTGGAG / TGGGCGACACAAACAGGT | Chr |
| F2241U / F2242L | RSc1921 | CGATGGTGACGGCAGAG / GATCGAGTGGTGGCAGAC | Chr |
| F2116U / F2117L | RSc2191 (*purD*) | GACCGAAGTCGAGTTCAC / AGTAGTCGTCGCACCACT | Chr |
| F2251U / F2252L | RSc2341 (*ftsK*) | TTCCAGGGATGCGGTAAC / CCCGAGCTTCCACCACT | Chr |
| F2243U / F2244L | RSc2585 (*tn*) | GGATTCACCGCCACTTCA / ATCCGCAGCTTCCTCATC | Chr |
| F2255U / F2256L | RSc3023 (*rpsG*) | CTAGCCTCGTCCCAAATG /CCCGCAGGTCTTCTCTC | Chr |
| F2249U / F2250L | RSc3252 | TAGGAACGGCCACAGTCA / CCTCGGCGTCTACAAGC | Chr |
| F1688U / F1689L | RSc3437 (*vsr*) | GTGGTGGGCACCGCTACA / CGTGGCGCTGGACCTGTC | Chr |
| F2094U / F2095L | RSp0313 (*mexC*) | GGATTGCAGGCCGATGAT / GCATGATGACGGGCGTAT | MP |
| F2245U / F2246L | RSp1328 | CCAGGCAGAGGCGACAC / GGCGGGAGTGGATGTTGT | MP |
| F0379U / F0380L | RSp0877 *(popA)* | CGCCGCCGAGCTGGGCGGAGG / CGCCCTGCGCCTGTTGT | MP |

^a^ Chr, Chromosome; MP, megaplasmid
