# Supplementary material for: Horizontal Gene Transfer Regulation in Bacteria as a “Spandrel” of DNA Repair Mechanisms
Source: PLoS One. 2007 Oct 24;2(10):e1055. doi: 10.1371/journal.pone.0001055 (PMC2013936; doi:10.1371/journal.pone.0001055)
Supplement: Table S3 — (0.08 MB DOC) [file pone.0001055.s003.doc]

**Table S3.** Characteristics of strains and plasmid used in this study

| Targeted position  accession number (gene acronym) | PCR product acronym^a^ | Initial cloning vector | Cloning site | Relevant properties | Plasmid acronym | Recombinant strain acronym |
| --- | --- | --- | --- | --- | --- | --- |
| RSc0171 | TCP 1 | pCR 2.1-TOPO | *Sma*I | pCR 2.1-TOPO, RSc0171::Gm | pTCP 1 | gTCP 1 |
| RSc0458 (*ubiE*) | TCP 2 | pGEM-T Easy | *Sma*I | pCR 2.1-TOPO, RSc0458::Gm | pTCP 2 | gTCP 2 |
| RSc0551 (*recA*) | TCP 3 | pUC19 | *BstX*I | pCR 2.1-TOPO, RSc0551::Gm | pTCP 3 | gTCP 3 |
| RSc0558 (*pilA*) | TCP 4 | pCR 2.1-TOPO | *SspI* | pCR 2.1-TOPO, RSc0558::Gm | pTCP 4 | gTCP 4 |
| RSc0828 (*tIS14b*) | TCP 5 | pCR 2.1-TOPO | *Sal*I | pCR 2.1-TOPO, RSc0828::Gm | pTCP 5 | gTCP 5 |
| RSc1120 (*comA-like*) | TCP 6 | pUC19 | *Bsp681* | pCR 2.1-TOPO, RSc1120::Gm | pTCP 6 | gTCP 6 |
| RSc1151 (*mutS*) | TCP 7 | pUC19 | *Sma*I | pCR 2.1-TOPO, RSc1151::Gm | pTCP 7 | gTCP 7 |
| RSc1815 | TCP 8 | pCR 2.1-TOPO | *Sma*I | pCR 2.1-TOPO, RSc1815::Gm | pTCP 8 | gTCP 8 |
| RSc1921 | TCP 9 | pCR 2.1-TOPO | *Sph*I | pCR 2.1-TOPO, RSc1921::Gm | pTCP 9 | gTCP 9 |
| RSc2191 (*purD*) | TCP 10 | pGEM-T Easy | *Xho*I | pCR 2.1-TOPO, RSc2191 ::Gm | pTCP 10 | gTCP 10 |
| RSc2341 (*ftsK*) | TCP 11 | pGEM-T | *Eco*RI | pCR 2.1-TOPO, RSc2341::Gm | pTCP 11 | gTCP 11 |
| RSc2585 (*Tn*) | TCP 12 | pGEM-T | *Eco*RI | pCR 2.1-TOPO, RSc2585::Gm | pTCP 12 | gTCP 12 |
| RSc3023 (*rpsG*) | TCP 13 | pGEM-T Easy | *Msc*I | pCR 2.1-TOPO, RSc3023 ::Gm | pTCP 13 | gTCP 13 |
| RSc3252 | TCP 14 | pCR 2.1-TOPO | *Ssp*I | pCR 2.1-TOPO, RSc3252::Gm | pTCP 14 | gTCP 14 |
| RSc3437 (*vsr*) | TCP 15 | pUC19 | *Pf*l23II | pCR 2.1-TOPO, RSc3437::Gm | pTCP 15 | gTCP 15 |
| RSp0313 (*mexC*) | TMP 1 | pGEM-T | *Xho*I | pCR 2.1-TOPO, RSp0313 ::Gm | pTMP 1 | gTMP 1 |
| RSc1328 | TMP 2 | pGEM-T | *Bgl*II | pCR 2.1-TOPO, RSp1328::Gm | pTMP 2 | gTMP 2 |
| RSp0877 (*popA*) | TMP 3 | pBluescript | *BamHI* | pCR 2.1-TOPO, RSp1328::Gm | pTMP 3 | gTMP 3 |

^a^C and M for chromosomal and megaplasmid positions, respectively
